# Medical nutrition therapy in non-alcoholic fatty liver disease – a review of literature

**Published:** 2015

**Authors:** E Rusu, G Enache, M Jinga, R Dragut, R Nan, H Popescu, C Parpala, C Homentcovschi, M Nitescu, M Stoian, A Costache, M Posea, F Rusu, V Jinga, D Mischianu, G Radulian

**Affiliations:** * “Carol Davila” University of Medicine and Pharmacy, Bucharest, Romania

**Keywords:** diet, caloric restriction, lifestyle changes, management, steatohepatitis

## Abstract

**Background and aim:** Non-alcoholic fatty liver disease (NAFLD) is the most common cause of liver disease worldwide, with a prevalence of 20%-40% in Western populations. The purpose of this article is to review data related to lifestyle changes in patients with NAFLD.

**Method:** We searched a public domain database (PubMed) with the following categories: disease (NAFLD, fatty liver, and non-alcoholic steatohepatitis [NASH]) and intervention (lifestyle intervention, diet, nutrition) with each possible combination through 25 September 2014, for relevant articles. Review of articles was restricted to those published in English. We selected the studies involving adult patients only.

**Conclusion:** There is no consensus as to what diet or lifestyle approach is the best for NAFLD patients. However, patients with NAFLD may benefit from a moderate- to low-carbohydrate (40%–45% of total calories) diet, coupled with increased dietary MUFA and n-3 PUFAs, reduced SFAs. More CRT are needed to clarify the specific effects of different diets and dietary components on the health of NAFLD patients.

**Abbreviations:** NAFL = Non-alcoholic fatty liver, NAFLD = non-alcoholic fatty liver disease, NASH = non-alcoholic steatohepatitis, HCC = hepatocarcinoma, BEE = basal energy expenditure, CRT = A small clinical randomized trial showed that short-term carbohydrate restriction is more efficacious in reducing intrahepatic triglyceride, IHT = intrahepatic triglyceride, VLCD = Very low calorie diets, AST = aspartate aminotransferases, SFAs = saturated fatty acids.

## Introduction

Non-alcoholic fatty liver (NAFL) is a part of the spectrum of liver disease called non-alcoholic fatty liver disease (NAFLD) [**1**]. The NAFLD includes steatohepatitis (NASH), fibrosis, cirrhosis and hepatocarcinoma (HCC).

NAFLD is the most common cause of liver disease worldwide, with a prevalence of 20%-40% in Western populations [**1**]. In Europe, the prevalence of NAFLD varies between 20-30% [**2**]. The prevalence increases to 58% in overweight individuals and can be as high as 98% in non-diabetic obese individuals [**3**]. 

## Methods

**Study selection**

A public domain database (PubMed) with the following categories was searched for relevant articles: disease (NAFLD, fatty liver, and non-alcoholic steatohepatitis [NASH]) and intervention (lifestyle intervention, diet, nutrition) with each possible combination through 25 August 2014. Review of articles was restricted to those published in English. Only the studies involving adult patients were selected. 

**Energy requirements**

In healthy individuals, energy requirements represent the number of calories that a person needs to achieve certain weight goals - meaning to lose, or maintain weight. Nutrition guidelines for population with NAFLD recommended a hypocaloric diet based on individual needs to promote weight loss [**4**] in a professional review, McCarthy and Rinella recommended 1200-1500 kcal/ day [**5**]. If liver disease progresses to cirrhosis, the calorie requirements increase even more to prevent undesired weight loss and malnutrition.

Energy requirements for patients with NAFLD are: 25 to 30 kcal/ kg, based on dry weight or an adjusted ideal weight, or add 20% to 40% to basal energy expenditure (BEE) by using the Harris-Benedict equation [**6**-**8**]. Energy deficit between 500 and 1000 kcal per day will lead to weight loss of 0.5–1 kg per week [**9**,**10**] but it should not exceed 1 kg per week. Rapid and uncontrolled weight loss can be detrimental for patients and may even worsen clinical symptoms of NAFLD [**11**]. Also, the risk of gallstone disease increases exponentially when the rate of weight loss exceeds 1.5 kg/ wk [**12**]. Very low calorie diets (VLCD) (388 kcal/ day) can cause the activation of overall inflammation and a rise in serum bilirubin levels [**13**]. In another study, Lewis MC et al. [**14**] investigated 18 morbidly obese subjects who underwent Optifast very low caloric diet (VLCD). After six weeks, subjects had a mean weight reduction of 9 kg and 43% reduction in mean liver fat.

In NASH patients, a dietary intervention and weight loss was associated with improvement in liver histology and enzymes [**15**]. 

Petersen KF et al. [**16**] showed that a low fat diet reduced calorie intake (daily intake 1200 kcal/ day) that effectively reduces body weight and intra-hepatic lipid content with improvement of insulin resistance in NAFLD patients. 

Promrat K et al. [**15**] conducted a small study (n=31) of patients randomly assigned to receive either diet, exercise, and behavioral strategies or a control group (2:1 ratio). Participants who achieved the study weight loss goal of 7% had significantly greater improvements in all aspects of NASH histologic activity including steatosis, lobular inflammation, ballooning injury [**15**].

Huang MA et al. [**17**] showed that a diet of 1400 kcal/ d could be effective in improving histology in patients with biopsy-proven NASH.

**Table 1:** Diet trials in NAFLD

• Daily 500-1000 calorie intake reduction [**9**,**10**,**18**]
• Daily 1200-1500 calorie intake [**5**,**17**]
• Restriction of caloric intake to 25-30 kcals/kg/day of ideal body weight [**6**-**8**]
• Restriction of total dietary fat content to <30% of the caloric intake with <10% of the caloric intake from saturated fats [**7**,**8**]
• Low calorie/ low carbohydrate (40–45% of caloric intake) [**17**]
• Very low calorie diet [**13**,**14**]

**Carbohydrates**

AMDR for carbohydrates in adults ranges between 45-65% of total number of calories [**19**]. The best carbohydrate sources are fruits, vegetables, whole grains, legumes and low-glycemic index foods [**19**].

Data from literature reports that a diet high in carbohydrates might worsen the clinical conditions of patients with NAFLD [**6**].

Valtueña S et al. [**20**] demonstrated a positive relationship between higher quartiles of dietary GI with higher grades of hepatic steatosis in a population of 247 apparently healthy adults.

Haufe S and colleagues [**21**] randomized 102 overweight and obese individuals to a 6-months dietary intervention consisting of either low-carbohydrate or low-fat hypocaloric diet, and observed improvements in intrahepatic lipid content in both groups with no significant differences.

In several studies, fructose was associated with NAFLD [**22**] but in a recent review, the relationship between fructose or sucrose intake and NASH appears to be confounded by excessive energy intake [**23**].

A small clinical randomized trial (CRT) showed that short-term carbohydrate restriction is more efficacious in reducing intrahepatic triglyceride (IHT) than caloric restriction [**24**].

Low percentage of calories from carbohydrates (40%) showed lower levels of alanine aminotransferase (ALT) [**25**].

In another study using two energy-restricted diets with equal energies, but different proportions of carbohydrates, showed similar weight loss but a greater decrease in triglycerides in the group with a lower proportion of carbohydrates [**26**].

Consumption of whole grains compared to refined grains is associated with the reduction of the abdominal fat mass [**27**-**29**]. For patients with NAFLD, decrease of body fat may be more important, supporting the use of whole grains as a carbohydrate source during hypocaloric diets.

**Fiber**

Requirements for general population are set at 38 g/ day for men and 25 g/ day for women aged 19 to 50 years [**30**]. In many countries, this now includes the recommendation to eat at least half of all grain servings as whole grains, with gram recommendations ranging from at least 48 g/ d to at least 75 g/ 2000 kcal [**31**].

**Lipids**

AMDR for lipids is 20% to 35% of the total energy intake for adults [**19**]. For n-6 PUFAs, the AMDR is 5% to 10% of energy, which is expected to meet the adequate intake (AI) for linolenic acid (17 g/ day for young men and 12 g/ day for young women). The AMDR for alpha linolenic acid is 0.6% to 1.2% of energy, with up to 10% of the AMDR consumed as eicosapentaenoic acid (EPA) and/ or docosahexaenoic acid (DHA); the AI is 1.6 g/ day for men and 1.1 g/ day for women. The DHA and EPA deficiency can contribute to the development of NAFLD, which was confirmed by an experimental study on animals [**19**].

McCarthy and Rinella’s review identified evidence to support negative effects of high (>10%) and low (<6%) dietary saturated fatty acids (SFAs) consumption, and suggested that a range of SFA between 6% and 10% may be most beneficial to patients with NAFLD, with an intake of MUFAs up to 25%, and increased intake of *n-3* PUFAs [**5**].

In Look AHEAD (Action for Health in Diabetes) trial [**19**] an intensive lifestyle intervention (moderate caloric restriction, <30% fat of total energy, increased physical activity, weight loss goal of 7% body weight) was associated with significant reductions in hepatic steatosis and HbA1c, but not in aspartate aminotransferases (AST) or ALT.

Westerbacka J et al. [**32**] showed that a high-fat diet (56% total energy) is associated with increased hepatic steatosis and serum insulin resistance, in comparison to a low-fat diet (16% total energy). Nevertheless, the high-fat group consumed significantly more saturated fat, which can be a confounding factor between the high-fat diet and hepatic steatosis [**32**].

In a study by Yamamoto M et al. [**33**] reduction of lipid consumption for 6 months induced a decrease in liver enzymes.

A diet with MUFAs over 40% of energy as fat was associated with decreased very-low-density lipoprotein (VLDL)-cholesterol and TG, and it was more acceptable to patients with diabetes than a high-carbohydrate diet [**34**]. 

Increasing in the intake of MUFAs and decreasing the amount of SFAs and carbohydrates in the diet may be beneficial for patients with NAFLD [**35**]. In rats, a diet rich in SFAs increased oxidative stress in mitochondria and contributed to the impair of hepatocytes [**36**].

Supplementation with omega-3, 2 g/ day for 6 months decreased the level of steatosis as well as serum ALT activity, and serum TG levels [**37**]. Hatzitolios A et al. demonstrated a decline in transaminase levels and normalization of ultrasonographic evidence of fatty liver on treatment with omega-3 fatty acids (15 ml/ day) in patients with hypertriglyceridemia and resolved fatty liver in 35% of patients [**38**].

Sofi F et al. [**39**] demonstrated that the long-term consumption of olive oil enriched with n-3 PUFAs lead to lower liver enzyme activities and TG levels.

Zhu FS et al. [**40**] reported that n-3 PUFAs from seal oils are safe and efficacious in hyperlipidemic patients with NAFLD, serum ALT activities, and normalize hepatic fat infiltration.

Oya J et al. [**41**] found that dietary EPA and EPA with DHA might be independent and preventive nutrients for NAFLD in Japanese men. EPA has shown to improve NASH, likely due to its antioxidative and anti-inflammatory properties [**42**].

**Protein**

Protein intake must be adjusted for body weight and medical condition. According to the most recent data, normal protein intake in adults is around 16–17% of energy intake [**43**]. The requirement of protein in hepatitis is estimated minimally at 1.0 to 1.2 g/kg/day and may range up to 1.5 g/ kg if patients have cirrhosis.

The AASLD Guidelines [**44**] do not specifically address protein intake, and recommends a protein intake similar to the general population, based on the recommended dietary allowances (RDA) and dietary reference intake (DRI).

## Conclusions

There is no consensus as to what diet or lifestyle approach is the best for NAFLD patients. However, patients with NAFLD may benefit from a moderate- to low-carbohydrate (40%–45% of total calories) diet, coupled with increased dietary MUFA and *n-3* PUFAs, reduced SFAs. More CRT are needed to clarify the specific effects of different diets and dietary components on the health of NAFLD patients.

**Conflicts of Interest**

The authors declare no conflict of interest.

**Acknowledgement:** This paper is supported by The Sectoral Operational Programme Human Resources Development (SOP HRD), financed from the European Social Fund and by the Romanian Government under the contract number POSDRU/159/1.5/S/137390
